# Is the vitamin D status of patients with COVID-19 associated with reduced mortality? A systematic review and meta-analysis

**DOI:** 10.20945/2359-3997000000588

**Published:** 2023-01-31

**Authors:** Paulo R. Bignardi, Paula de Andrade Castello, Bruno de Matos Aquino, Vinicius Daher Alvares Delfino

**Affiliations:** 1 Pontifícia Universidade Católica do Paraná Faculdade de Medicina Londrina PR Brasil Faculdade de Medicina, Pontifícia Universidade Católica do Paraná, Londrina, PR, Brasil.; 2 Universidade Estadual de Londrina Londrina PR Brasil Universidade Estadual de Londrina, Londrina, PR, Brasil.

**Keywords:** 1,25-dihydroxyvitamin D, 25-hydroxyvitamin D, meta-analysis, systematic review, SARS-CoV-2

## Abstract

To conduct a systematic review and meta-analysis of studies assessing the association between serum vitamin D status and mortality in patients with COVID- 19. We searched PubMed and Embase for studies addressing the association of serum vitamin D levels and COVID-19 mortality published until April 24, 2022. Risk ratios (RRs) and 95% confidence interval (CIs) were pooled using fixed or random effects models. The risk of bias was assessed using the Newcastle-Ottawa Scale. The meta-analysis included 21 studies that measured serum vitamin D levels close to the date of admission, of which 2 were case- control and 19 were cohort studies. Vitamin D deficiency was associated with COVID-19 mortality in the overall analysis but not when the analysis was adjusted to vitamin D cutoff levels < 10 or < 12 ng/mL (RR 1.60, 95% CI 0.93-2.27, I2 60.2%). Similarly, analyses including only studies that adjusted measures of effect for confounders showed no association between vitamin D status and death. However, when the analysis included studies without adjustments for confounding factors, the RR was 1.51 (95% CI 1.28-1.74, I2 0.0%), suggesting that confounders may have led to many observational studies incorrectly estimating the association between vitamin D status and mortality in patients with COVID-19. Deficient vitamin D levels were not associated with increased mortality rate in patients with COVID-19 when the analysis included studies with adjustments for confounders. Randomized clinical trials are needed to assess this association.

## INTRODUCTION

Since the first case of COVID-19 was recorded in Wuhan (Hubei Province, China) in December 2019, SARS-CoV-2 infection has spread rapidly across the globe due to the strong contagious and infectious characteristics of this virus ( 1 , 3 ). Until May 13, 2022, COVID-19 has caused 6,216,708 deaths ( 4 ).

Global data from the pandemic has demonstrated a mortality rate of 0.9% in patients with COVID-19 and without comorbidities, but this rate grows progressively with the patients’ increasing age and number of comorbidities ( 5 ). Studies associating serum vitamin D levels with acute respiratory infections ( 6 ) have led to an ecological study, which showed that countries with populations with lower serum vitamin D levels have higher infection rates and mortality associated with SARS-CoV-2 infection ( 7 ). Additionally, Isaia and cols. ( 8 ) have found a correlation between higher levels of solar ultraviolet (UV) radiation in some regions and lower morbidity and mortality rates related to COVID-19. The authors hypothesized that this finding could be related to the vitamin D levels in the population.

Skin exposure to UV radiation determines the local photoconversion of 7-dehydrocholesterol to previtamin D3 ( 9 ). Two forms of vitamin D are available, *i.e.* , D2 and D3, and the primary source of vitamin D3 – which comprises about 80% of the vitamin D stored in the body – derives from UV conversion ( 10 ). Vitamin D formed in the skin or obtained from diet is metabolized in the liver into 25-hydroxyvitamin D (25[OH]D), which is the vitamin form used to determine a patient's vitamin D status. This form is then hydroxylated in the kidneys into the active form of the vitamin, *i.e.* , 1,25 dihydroxyvitamin D (1,25[OH]_2_D).

Vitamin D status is defined according to 25(OH)D levels as insufficient when < 30 ng/mL, deficient when < 20 ng/mL, and severely deficient when < 10 ng/mL ( 11 , 12 ) or <12 ng/mL, according to some authors ( 13 , 14 ). Small observational studies analyzing the association of vitamin D deficiency or insufficiency with COVID-19 outcomes have shown conflicting results ( 15 , 19 ). Based on these considerations, we conducted a systematic review and meta-analysis to assess the association between vitamin D status and mortality in patients with COVID-19.

## METHODS

This systematic review was conducted following the Preferred Reporting Items for Systematic Reviews and Meta-Analyses (PRISMA). This study has not been registered.

### Data search

In November 2020, two of the investigators performed a search in PubMed, Embase, and Cochrane Library databases. Studies published until April 24, 2022, were included in the analysis. The following search strategy was used: “vitamin D” AND “coronavirus” OR “coronavirus infections” OR “COVID-19” OR “severe acute respiratory syndrome coronavirus 2”. Among the articles identified in the search, only those published in English, Spanish, or Portuguese were included in the analysis.

### Study selection

Two independent authors screened the retrieved arti- cles. Disagreements were resolved through discussion among all the authors. The authors read the abstracts of the retrieved articles and, after excluding irrelevant studies, proceeded to read the full article for screening.

For inclusion in the analysis, the studies should meet the following population, intervention, control, and outcomes (PICO) criteria: (A) include hospitalized patients with COVID-19 (must have included throat swabs for direct identification of upper respiratory SARS-CoV-2 infection using nucleic acid real-time reverse transcription-polymerase chain reaction [RT- PCR] to confirm the diagnosis of COVID-19); (B) include patients with COVID-19 older than 18 years and with vitamin D status identified as “deficient” or “insufficient” by measurement of serum vitamin D (25[OH]D) levels close to the date of the COVID-19 diagnosis (specifically, up to 30 days before or up to 7 days after the diagnosis); (C) enroll patients with COVID-19 and vitamin D status identified as “sufficient,” who were used for comparison; (D) examined the association between vitamin D status and mortality. Studies in which serum vitamin D levels measured at admission were not mentioned, letters to the editor, case reports, and articles reporting ecological, cross-sectional, animal model, or pediatric studies were excluded from the analysis.

### Data extraction

The eligible studies included the assessment of death in individuals with measured serum vitamin D levels. The studies should have informed odds ratios (ORs), risk ratios (RRs), or hazard ratios (HRs) with 95% confidence intervals (CIs). The inclusion in the analysis was not restricted by study size.

The data extracted from the studies included information about the authors, study design, country of origin, patients’ demographic characteristics (age and sample size), COVID-19 diagnosis, outcomes, and confounders. Two independent investigators extracted the data using a structured form designed by the authors. Disagreements were resolved through discussion among all authors.

### Analysis of results

The analysis focused on the impact of vitamin D deficiency on mortality outcomes in patients with COVID-19. For this, we first performed an analysis stratified by vitamin D cutoff level (< 20 or < 25 ng/mL and < 10 or < 12 ng/mL, according to each study). We then performed another stratified analysis including studies that adjusted analyses for confounding factors versus those that did not perform such adjustments. For the present meta-analysis, we standardized the vitamin D measurement unit as ng/mL, and for those studies with levels presented in nmol/L, we transformed the values into ng/mL ( 10 ). We also performed sensitivity analysis by omitting individual studies to detect the influence of each study on the overall effect estimate.

### Quality assessment, risk of bias, and statistical analysis

The Newcastle-Ottawa Scale was used to evaluate the quality of observational studies. This scale assigns up to 9 points to each study (4 points for selection, 2 points for comparability, and 3 points for exposure or outcome) ( 20 ). Studies scoring at least 6 points are considered to have a low risk of bias. The studies included in the present meta-analysis reported ORs, HRs, or RRs; for studies not reporting these effects, the RR was calculated according to the Cochrane Handbook for Systematic Reviews of Interventions ( 21 ).

We extracted the effect estimates with the most significant degree of adjustment for potential confounding factors. We also considered HR comparable to RR. For studies reporting ORs, a corrected RR was computed as previously described ( 22 ). Pooled RRs and 95% CIs were calculated using fixed or random effects models according to the study's homogeneity. Cochran's Q test and I^2^ statistic were used to evaluate, respectively, the statistical significance and the degree of heterogeneity between the studies. An I^2 ^statistic value ≥ 50% reveals substantial heterogeneity. Sensitivity analysis was also performed, removing each study from the meta-analysis to investigate the source of heterogeneity. Finally, publication bias was examined using Egger's test and funnel plot analysis. All analyses were performed using the software Stata/SE, v.14.1 (StataCorp LLC, College Station, TX, USA).

## RESULTS

### Characteristics of the selected studies

**Figure 1:**
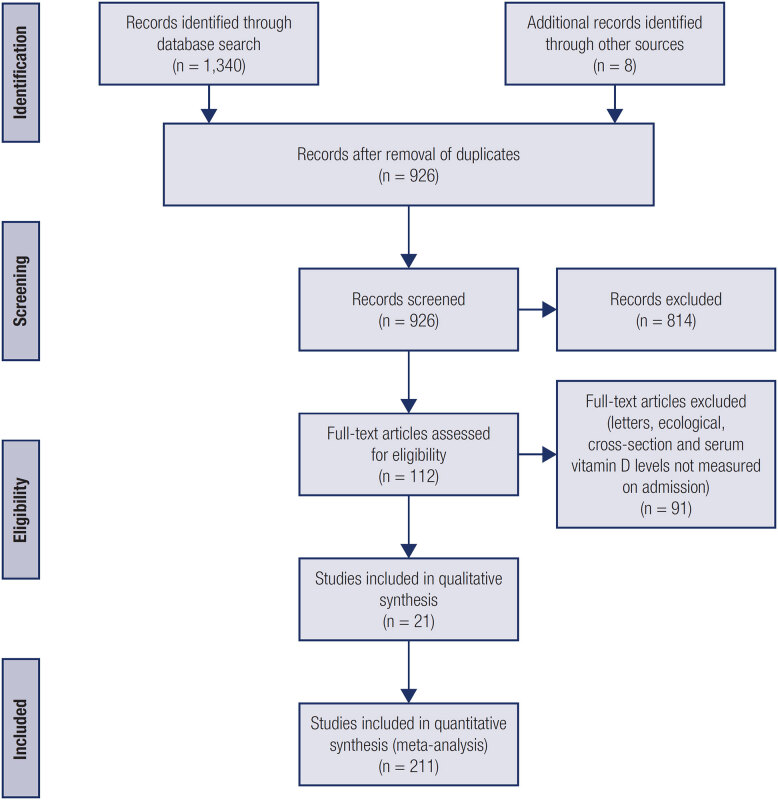
Flowchart of study selection.

The database search identified 1,340 articles. Of these, 814 were duplicates or were excluded based on predetermined eligibility criteria during title/ abstract review. After applying the inclusion and exclusion criteria, we identified 21 articles ( 23 , 43 ) eligible for the present systematic review and meta- analysis ( Figure 1 ), which involved 6,096 participants. Among the identified articles, 19 were cohort studies ( 23 , 24 , 26 – 28 , 30 – 34 , 36 – 43 ) and two were case-control studies ( 25 , 35 ). Table 1 summarizes the characteristics of the selected studies and study participants.

**Table 1:** Characteristics of the selected studies

Author	Country	Study Design	Follow-up	Population	Age (years) *	Outcomes	Sample Size	Exposure, n (cutoff level)	Adjusted confounders
Abrishami and cols., 2021 ( 23 )	Iran	Retrospective cohort	February 28 2020 – April 19, 2020	Patients hospitalized with COVID-19	55.18 ± 14.98	Death and hospitalization	73	-Vitamin D≤ 25 ng/mL	Age, sex, and comorbidities
Afaghi and cols., 2021 ( 39 )	Iran	Retrospective cohort	March 16 2020–February 25, 2021	Patients hospitalized with COVID-19	53.7 ± 15.8	ICU admission, invasive mechanical ventilation, duration of hospitalization, and death	646	109(Vitamin D≤ 20 ng/mL)	Age, sex, and comorbidities
AlSafar and cols., 2021 ( 38 )	United Arab Emirates	Retrospective cohort	August 2020 –February 2021	Adult patients with COVID-19	46.6 ± 14.6	Death and severity	464	127 (Vitamin D≤ 12 ng/mL)182(Vitamin D≤ 20 ng/mL)	Age, sex, smoking status, and comorbidities
Baktash and cols., 2020 ( 24 )	UK	Prospective cohort	March 1^st^–April 302020	Patients aged ≥ 65 years hospitalized with COVID-19	81 (65-102) *	Mortality secondary to COVID-19; NIV support and admission to HDU, COVID-19 radiographic changes on chest X-ray	70	39 (Vitamin D≤ 12 ng/mL)	Not adjusted
Barassi and cols., 2021 ( 33 )	Italy	Prospective cohort	April 8 –May 25 2020	Patients hospitalized with COVID-19	61 (24-92) *	Mortality	118	87 (Vitamin D≤ 20 ng/mL)	Not adjusted
Bennouar and cols., 2021 ( 30 )	Algeria	Prospective cohort	July 6 – August 15, 2020	Patients with severe COVID-19	62.3 ± 17.6	Mortality	120	32 (Vitamin D≤ 10 ng/mL)35 (Vitamin D≤ 20 ng/mL)	Age, sex, acute kidney injury, cardiac injury, blood glucose, CRP, NLR, LDH, albumin, and total cholesterol
Cereda and cols., 2021 ( 31 )	Italy	Cohort	March –April 2020	Patients hospitalized with COVID-19	77 (65-85) *	Mortality	129	99(Vitamin D< 20 ng/mL)	Age, CRP, and ischemic heart disease
Derakhshanian and cols., 2021( 41 )	Iran	Retrospective cohort	February 20 2020–April 20, 2020	Patients hospitalized with COVID-19	61.6 ± 16.9	COVID-19 severity (death and ICU admission)	290	142 (Vitamin D < 20 ng/mL)	Not adjusted
Güven & Gültekin 2021( 43 )	Turkey	Retrospective cohort	March 1^st^–December 31^st^, 2020	Patients hospitalized with COVID-19	64 (53-77) **	Survival	520	355 (Vitamin D< 12 ng/mL)	Age, sex, and comorbidities
Hafez and cols., 2022 ( 34 )	United Arab Emirates	Retrospective cohort	April – May 2020	Patients hospitalized with COVID-19	43 ± 12	Severity	126	10 (Vitamin D < 10 ng/mL)62(Vitamin D < 20 ng/mL)	Age, sex, race, and comorbidities
Hernández and cols., 2021( 25 )	Spain	Case-control	March 10 –March 31^st^, 2020	Patients hospitalized with COVID-19	61 (47.5-70) **	COVID-19 severity (death, ICU admission, and NIV)	216	162 (Vitamin D ≤ 20 ng/mL)	Age, sex, BMI, smoking, diabetes mellitus, history of cardiovascular events, oral vitamin D supplements, CRP, and GFR
Infante and cols., 2021 ( 40 )	Italy	Retrospective cohort	March 1^st^ –April 302020	Patients hospitalized with COVID-19	Survivors 65 ± 13 Non-survivors 70 ± 29	Survival, length of stay in hospital and ICU	137	69 (Vitamin D ≤ 10 ng/mL)	Not adjusted
Jain and cols., 2020 ( 26 )	India	Prospective cohort	6 weeks	Patients with COVID-19 aged 30-60 years	51.41 ± 9.12	Serum IL-6, serum TNF-alpha, serum ferritin, deaths, and serum level of vitamin D	154	90 (Vitamin D< 20 ng/mL)	Not adjusted
Karahan & Katkat, 2021 ( 29 )	Turkey	Retrospective cohort	April 1^st^–May 20 2020	Adult patients with moderate to severe COVID-19	63.5 ± 15.3	Mortality	149	103 (Vitamin D < 20 ng/mL)	Age, smoking, hyperlipidemia, diabetes mellitus, chronic kidney disease, chronic atrial fibrillation, congestive heart failure, acute kidney injury, eGFR, hemoglobin, neutrophil count, lymphocyte count, white blood cell count, CRP, albumin, and calcium
Nimavat and cols., 2021 ( 35 )	India	Case-control	August 1^st^–September 5, 2020	Patients aged ≥18 years hospitalized with confirmed COVID-19	41.6 ± 16.4	Mortality	156	25 (Vitamin D < 10 ng/mL)	Not adjusted
Pecina and cols., 2021( 36 )	USA	Retrospective cohort	April 16 –October 17, 2020	Patients aged ≥18 years hospitalized with confirmed COVID-19	61 (50-74) **	ICU mortality and duration of hospitalization	92	15(Vitamin D < 20 ng/mL)	Not adjusted
Radujkovic and cols., 2020 ( 27 )	Germany	Prospective cohort	March 18 –June 18 2020	Inpatients and outpatients diagnosed with COVID-19	60 (49-70) **	NVI and survival	93	29 (Vitamin D< 12 ng/mL) 47(Vitamin D < 20 ng/mL)	Age, sex, and presence of comorbidity
Ramirez- Sandoval and cols., 2022 ( 42 )	Mexico	Retrospective cohort	March 13 2020 –March 1^st^, 2021	Patients with severe COVID-19	57 (46-67) **	Survival and discharge	2098	571 (Vitamin D< 12 ng/mL)	Age, sex, BMI, acute kidney injury, and diabetes
Reis and cols., 2021 ( 37 )	Brazil	Prospective cohort	June 2^nd^ July 21^st^, 2020, and July 22^nd^ September 25^th^, 2020	Patients aged ≥18 years hospitalized with confirmed COVID-19	Vitamin D <10 ng/mL group: 61.3 ± 14.4 Vitamin D ≥10 ng/mL group: 54.7 ± 14.5	Mortality and duration of hospitalization	220	16 (Vitamin D < 10 ng/mL)	Not adjusted
Smet and cols., 2020 ( 28 )	Belgium	Retrospective cohort	March 1^st^–April 7, 2020	Patients hospitalized with COVID-19	69 (52-80) **	COVID-19 severity (stage disease and death)	186	109 (Vitamin D < 20 ng/mL)	Age, sex, coronary artery disease, and diabetes
Vassiliou and cols., 2021 ( 32 )	Greece	Prospective cohort	March 18 –May 25 2020	Patients hospitalized with COVID-19	61 ± 14	Mortality and NIV	39	32 (Vitamin D < 20 ng/mL)	Not adjusted

Abbreviations – BMI: body mass index, CRP: C-reactive protein; GFR: glomerular filtration rate; eGFR: estimated glomerular filtration rate; HDU: high-dependency unit; TNF-alpha: tumor necrosis factor alpha; IL interleukin 6; ICU: intensive care unit; LDH: lactate dehydrogenase; NLR: neutrophil to lymphocyte ratio; NIV: noninvasive ventilation.

* Data represented as mean ± standard deviation.

** Data represented as median (interquartile range).

### Serum vitamin D level and mortality in patients with COVID-19

**Figure 2:**
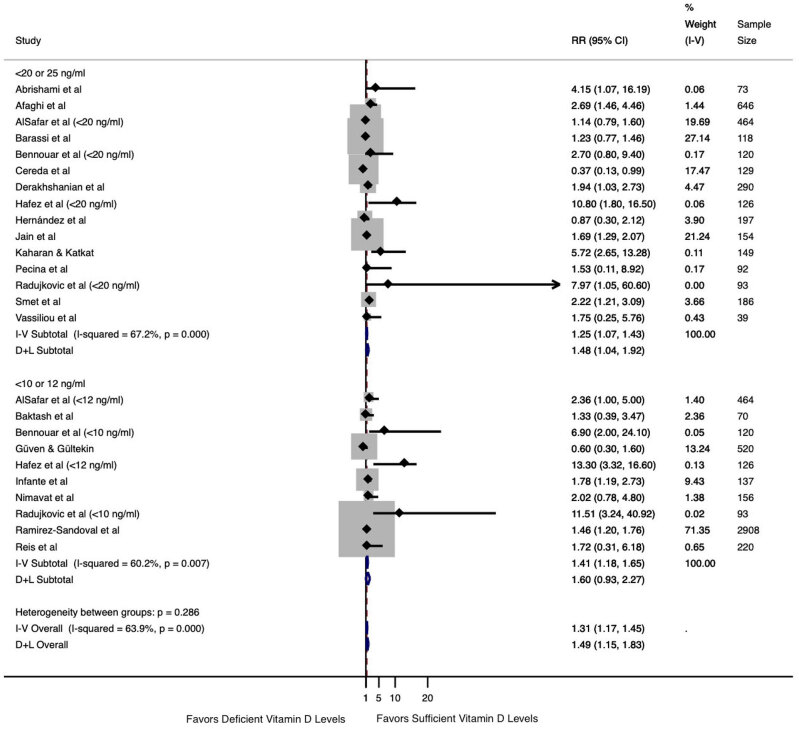
Association between serum vitamin D levels and mortality in patients with COVID-19 according to 25(OH)D cutoff level.

The mortality outcome was extracted from all 21 studies. The studies by AlSafar and cols. ( 38 ), Bennouar and cols. ( 30 ), Hafez and cols. ( 34 ), and Radujkovic and cols. ( 27 ) were included twice as they analyzed two vitamin D cutoff levels (< 10 or < 12 ng/mL and < 20 ng/mL). As shown in Figure 2 , the overall mortality analysis differed between patients with deficient vitamin D levels (regardless of cutoff level adopted) versus those with sufficient vitamin D levels (RR 1.49, 95% CI 1.15-1.83, I^2^ 63.9%). Similarly, the analysis including the cutoff levels of < 20 or < 25 ng/mL indicated an increased risk of death (RR 1.48, 95% CI 1.04-1.92, I^2^ 67.2%). However, the analysis including the cutoff levels of < 10 or < 12 ng/mL showed no difference between the groups with deficient versus sufficient vitamin D levels (RR 1.60, 95% CI 0.93-2.27, I^2^ 60.2%).

**Figure 3:**
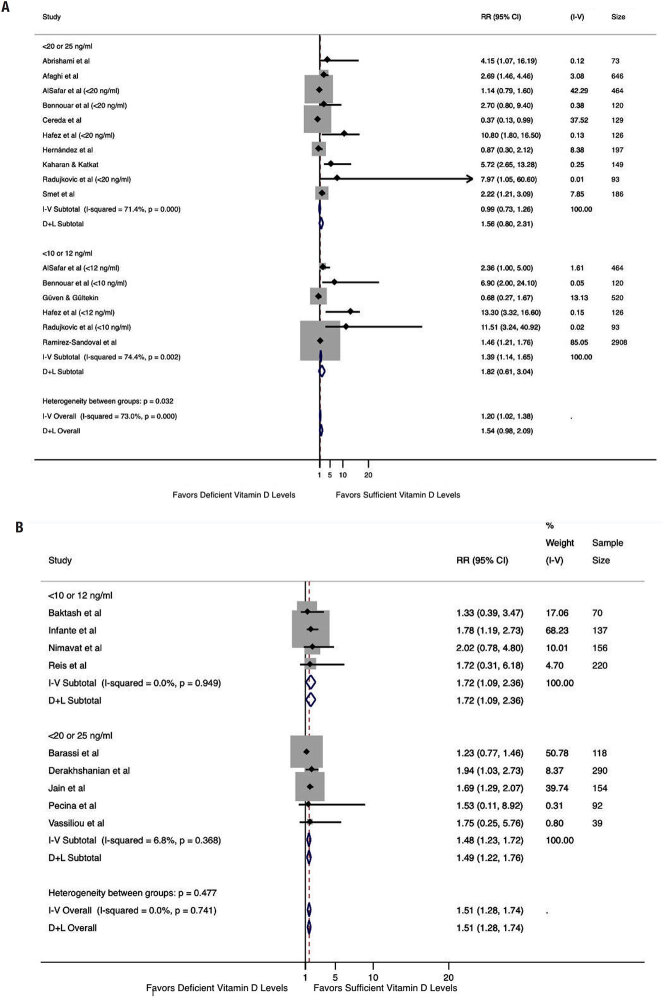
Association between serum vitamin D levels and mortality in patients with COVID-19, including studies that adjusted the analysis for age and at least one more confounder (obesity, hypertension, diabetes, renal disease, and cardiovascular disease) and studies without adjustment for confounders. (A) Analysis performed by 25(OH)D cutoff level, including only studies with adjustments; (B) analysis performed by 25(OH)D cutoff level, including studies without adjustments.

Subgroup analysis ( Figure 3A ) including studies performing analyses adjusted for age and at least one more confounding factor (obesity, hypertension, diabetes, chronic kidney disease, or cardiovascular disease) revealed no association between low vitamin D levels and death (RR 1.82, 95% CI 0.61-3.04, I^2^ 74.4% for cutoff levels of < 10 or < 12 ng/mL and RR 1.56, 95% CI 0.80-2.31, I^2^ 71.4% for cutoff levels of < 20 or < 25 ng/mL). In contrast, the analysis including studies not mentioning adjustment for confounders ( Figure 3B ), showed an increased risk of death for low vitamin D levels (RR 1.72, 95% CI 1.09-2.36, I^2^ 0.0% for cutoff levels of < 10 or < 12 ng/mL and RR 1.48, 95% CI 1.23-1.72, I^2^ 6.8% for cutoff levels of < 20 or < 25 ng/mL).

### Sensitivity analyses, assessment of heterogeneity, and risk of bias

Sensitivity analysis was performed excluding, individually, each study that adjusted for confounding factors regarding the mortality outcome. The Cochran's Q test remained unchanged, and the I2 varied from 44.1%-78.5% for the cutoff levels of < 10 or < 12 ng/mL and 56.7%-74.5% for the cutoff levels of < 20 or < 25 ng/mL, indicating that the result was not influenced by heterogeneity ( Table 2 ).

**Table 2:** Sensitive analysis for the mortality outcome, including studies that performed adjusted analysis for confounders

Study omitted (25[OH]D < 10 or < 12 ng/mL)	RR	95% CI	I^2^	p value *
AlSafar and cols., 2021	1.76	0.33-3.18	78.5%	>0.05
Bennouar and cols., 2021	1.77	0.54-2.99	78.5%	>0.05
Güven & Gültekin 2021	3.90	0.96-6.90	73.2%	>0.05
Hafez and cols., 2022	1.30	0.62-1.98	44.1%	>0.05
Radujkovic and cols., 2020	1.78	0.57-2.99	78.3%	>0.05
Ramirez-Sandoval and cols., 2022	3.99	0.66-7.23	77.8%	>0.05
**Study omitted (25[OH]D < 20 or < 25 ng/mL)**				
Abrishami and cols., 2021	1.54	0.78-2.30	74.0%	>0.05
Afaghi and cols., 2021	1.37	0.60-2.13	69.7%	>0.05
AlSafar and cols., 2021	1.95	0.81-3.09	73.9%	>0.05
Bennouar and cols., 2021	1.53	0.76-2.31	74.1%	>0.05
Cereda and cols., 2021	1.90	0.98-2.85	56.7%	>0.05
Hafez and cols., 2022	1.41	0.73-2.09	67.5%	>0.05
Hernández and cols., 2021	1.78	0.88-2.69	74.5%	>0.05
Kaharan & Katkat, 2020	1.46	0.73-2.20	71.9%	>0.05
Radujkovic and cols., 2020	1.56	0.80-2.33	74.4%	>0.05
Smet and cols., 2020	1.37	0.58-2.15	67.2%	>0.05

Abbreviation: 25(OH)D: 25-hydroyvitamin D.

* Value for heterogeneity among studies assessed with Cochran's Q test.

**Figure 4:**
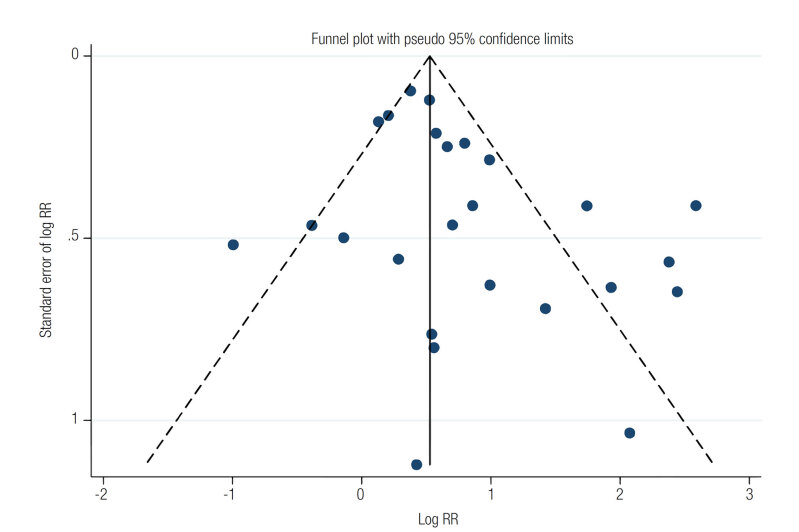
Funnel plot using data from 21 studies associating serum vitamin D levels and mortality. Four studies were included twice, as they analyzed two 25(OH)D cutoff levels.

Table 3 shows the risk of bias in the analyzed studies. The median quality score of the studies was 8 (range 6-9). The estimated bias coefficient was 0.105, with a *p* value of 0.053, indicating the absence of small- study effects. The funnel plot analysis performed also detected no possible small-study effects ( Figure 4 ). Therefore, the tests provided weak evidence for the presence of publication bias.

**Table 3:** Quality assessment of the selected articles according to the Newcastle-Ottawa Scale

Case-control	Selection				Comparability		Exposure			Total points
Studies	1	2	3	4	1A	1B	1	2	3	
Hernández and cols., 2021	Y	Y	Y	Y	Y	Y	N	Y	Y	8
Nimavat and cols., 2021	Y	Y	Y	Y	Y	N	Y	Y	Y	8
**Cohort**	**Selection**				**Comparability**		**Outcome**			**Total points**
**Studies**	1	2	3	4	1A	1B	1	2	3	
Abrishami and cols., 2021	Y	Y	Y	Y	Y	Y	Y	Y	Y	9
Afaghi and cols., 2021	Y	Y	Y	Y	Y	Y	Y	Y	Y	9
AlSafar and cols., 2021	Y	Y	Y	N	Y	Y	Y	Y	Y	8
Baktash and cols., 2020	Y	Y	Y	Y	Y	N	Y	Y	Y	8
Barassi and cols., 2021	Y	Y	Y	Y	Y	N	Y	Y	Y	8
Bennouar and cols., 2021	Y	Y	Y	Y	Y	Y	Y	Y	Y	9
Cereda and cols., 2021	Y	Y	Y	Y	Y	Y	Y	Y	Y	9
Derakhshanian and cols., 2021	Y	Y	Y	N	N	N	Y	Y	Y	6
Güven & Gültekin 2021	Y	Y	Y	Y	Y	Y	Y	Y	Y	9
Hafez and cols., 2022	Y	Y	Y	Y	Y	Y	Y	Y	Y	9
Infante and cols., 2021	Y	Y	Y	Y	N	N	Y	Y	Y	7
Jain and cols., 2020	Y	Y	Y	Y	N	N	Y	Y	Y	7
Karahan & Katkat, 2021	Y	Y	Y	Y	Y	Y	Y	Y	Y	9
Pecina and cols., 2021	Y	N	Y	Y	Y	Y	Y	Y	Y	8
Radujkovic and cols., 2020	Y	Y	Y	Y	Y	Y	Y	Y	Y	9
Ramirez-Sandoval and cols., 2022	Y	Y	Y	Y	Y	Y	Y	Y	Y	9
Reis and cols., 2021	Y	Y	Y	Y	N	Y	Y	Y	Y	8
Smet and cols., 2020	Y	Y	Y	Y	Y	Y	Y	Y	Y	9
Vassiliou and cols., 2021	N	Y	Y	Y	Y	N	Y	Y	Y	7

Abbreviations – N: no; Y: yes.

## DISCUSSION

This systematic review and meta-analysis assessed only studies in which serum vitamin D levels were measured close to the date of the COVID-19 diagnosis. Observational studies have associated low serum vitamin D levels and poor outcomes in patients with COVID-19. However, many of these studies used the level measured between a few months and many years before the diagnosis of COVID-19 ( 44 , 45 ). Vitamin D has known biological variability, and its serum levels can vary from 13%-26% over 4 months. The levels can also vary with age and with the emergence of comorbidities ( 46 , 50 ).

Despite the overall analysis and the analysis by cutoff levels of < 20 or < 25 ng/mL showing an association between low serum vitamin D levels and mortality, the analysis by cutoff levels of < 10 or < 12 ng/mL did not reveal increased mortality risk. A possible explanation for the association between mortality and vitamin D deficiency for the < 20 or < 25 ng/mL group but not for the < 10 or < 12 ng/mL group in the meta-analysis lies in the weight of the studies that did not adjust their analyses for confounders (54.8% in the < 20 or < 25 ng/mL group versus 4.4% in the < 10 or < 12 ng/mL group), as discussed below. The subgroup analyses including studies that have adjusted the analysis for age and at least one more confounder did not show such association, both in the overall analysis and in the analysis by cutoff levels. However, one analysis including only studies not adjusting for confounders revealed an association between low serum vitamin D levels and an increased (1.51 times) risk of death, which suggests that confounding factors may have driven many results in previous studies. Therefore, our meta- analysis suggests that vitamin D status has no causal effect on mortality in patients with COVID-19.

Investigating the causality of the association between vitamin D status and severity of COVID-19 infection, Patchen and cols. studied single nucleotide polymorphisms (SNPs) related to the risk of vitamin D deficiency. The authors found no association between genetically predicted differences in long-term vitamin D nutritional status and poor outcomes in patients with COVID-19 ( 51 ).

Hypovitaminosis D shares many risk factors with the severe form of COVID-19. Indeed, older age, obesity, chronic kidney disease, diabetes, hypertension, and cardiovascular disease are some critical risk factors that have been reported as associated with the severity of COVID-19 ( 52 , 55 ). Evidence indicates that vitamin D deficiency can be caused by older age, obesity, and chronic kidney disease ( 56 , 61 ). Type 2 diabetes, hypertension, and cardiovascular disease also are associated with vitamin D deficiency ( 62 , 63 ). Thus, many confounders may compromise the analysis between serum vitamin D levels and COVID-19 outcomes, and the results of observational studies must be interpreted with caution. Despite the well-known modulatory role of vitamin D in the immune response, the participation of the vitamin in preventing mortality in patients with COVID-19 may be outweighed by other mechanisms involved in the complex pathophysiology of the disease ( 64 , 65 ).

Other hypotheses must also be considered to explain our results. The liver hydroxylates vitamin D into 25(OH)D (calcifediol), the circulating form of the vitamin, which has a plasma half-life of 3 weeks (the whole-body half-life of this form is 2-3 months) ( 66 ). The serum calcifediol levels are generally used to check an individual's vitamin D status. Calcifediol is hydroxylated into its active form – *i.e.* , 1,25(OH)_2_D–which has a plasma half-life of 4 hours; this half-life is related to the presence of this form of the vitamin mainly in the kidneys but also in some sites other than the kidneys, including pulmonary epithelial cells ( 10 , 66 ). The serum level of 1,25(OH)_2_D is roughly 1,000 times lower than that of calcifediol ( 67 ). The kidney is the main organ regulating serum 1,25 (OH)_2_D ( 68 ). The ACE2 protein, which is present in renal and lung cells, is a target for SARS-CoV-2 to enter these cells ( 69 , 70 ). Renal dysfunction caused directly by the virus or indirectly by the presence of acute renal injury associated with COVID-19 infection may lead to a reduction in the activity of 1-alpha hydroxylase, the enzyme that converts calcifediol to 1,25(OH)2D ( 71 , 72 ).

Another possible hypothesis to explain our findings is that tissue damage caused by SARS-CoV-2 in the kidneys and, to a lesser extent, in the lungs may lead to an active decrease in 1,25(OH)D levels, which are responsible for the majority of the biological actions of vitamin D, but does not result in decreased levels of the vitamin form usually measured in serum (calcifediol). Previous studies have shown that serum 25(OH)D levels may not correlate with serum 1,25(OH)_2_D levels in some clinical conditions ( 73 , 75 ).

Reduced calcium and phosphorus have been found in critically ill patients with COVID-19, which may indicate a reduction in 1,25(OH)_2_D in these patients since the active form of vitamin D is an essential regulator of calcium and phosphorus levels acting in intestinal absorption and renal reabsorption ( 68 ).

Despite the well-known autocrine and paracrine production of 1,25(OH)_2_D by immune system cells, it is difficult to identify whether serum 1,25(OH)_2_D levels influence the immune response against SARS- CoV-2. Playford and cols. have shown that serum levels of 1,25(OH)_2_D but not those of 25(OH)D are associated with cardiovascular risk factors, a common outcome in patients with severe COVID-19 ( 76 ). Likewise, Nguyen and cols. have found that serum 1,25(OH)_2_D levels are a better predictor of mortality by sepsis than those of calcifediol ( 77 ). Notably, an antiviral action has been proposed for 1,25(OH)_2_D against SARS-CoV-2 and other viruses ( 78 , 79 ).

The present study has several strengths. To our knowledge, this is the first meta-analysis of studies that have included the vitamin D status from serum 25(OH)D levels measured on or close to the date of hospital admission in patients with COVID-19 and the first to include studies with analyses adjusted for confounders. The main limitations of the present study were the fact that the analysis included different cutoff levels for serum vitamin D in the same subgroup, that vitamin D levels were measured 30 days before hospitalization for COVID-19 in one of the included studies ( 43 ), the presence of substantial heterogeneity, and the observational design of the selected studies.

In conclusion, the results of the present study showed that, overall, vitamin D status was associated with mortality in patients with COVID-19, but not when the analysis included studies that adjusted measures of effect for confounding factors. Confounders may have led to the conclusion of the detrimental effects of low serum 25(OH)D levels in patients with COVID-19 observed in some previous studies. Large randomized clinical trials are needed to assess the effects of vitamin D levels (including those of 1,25[OH]_2_D) on mortality in patients hospitalized with COVID-19.
